# Neuromyotonia and CASPR2 Antibodies: Electrophysiological Clues to Disease Pathophysiology

**DOI:** 10.3390/biom15091262

**Published:** 2025-09-01

**Authors:** João Moura, Pietro Antenucci, Ester Coutinho, Kailash P. Bhatia, Lorenzo Rocchi, Anna Latorre

**Affiliations:** 1 Department of Neurology, Centro Hospitalar Universitário de Santo António, Unidade Local de Saúde de Santo António, 4099-001 Porto, Portugal; moura.neuro@chporto.min-saude.pt; 2 Unit for Multidisciplinary Research in Biomedicine (UMIB), School of Medicine and Biomedical Sciences (ICBAS), University of Porto, Rua Jorge Viterbo Ferreira 228, 4050-313 Porto, Portugal; 3 ITR—Laboratory for Integrative and Translational Research in Population Health, Rua das Taipas 135, 4050-600 Porto, Portugal; 4 Unit of Clinical Neurology, Neurosciences and Rehabilitation Department, University of Ferrara, Via Aldo Moro 8, Cona, 44121 Ferrara, Italy; pietro.antenucci@edu.unife.it; 5 Center for Neuroscience and Cell Biology, University of Coimbra, Rua Larga, 3004-504 Coimbra, Portugal; mcoutinho@uc.pt; 6 Centre for Innovative Biomedicine and Biotechnology (CiBB), University of Coimbra, Rua Larga, 3004-504 Coimbra, Portugal; 7 Católica Medical School, Universidade Católica Portuguesa, Estr. Octávio Pato, 2635-631 Rio de Mouro, Portugal; 8 Department of Clinical and Movement Neurosciences, UCL Queen Square Institute of Neurology, 33 Queen Square, London WC1N 3BG, UK; k.bhatia@ucl.ac.uk (K.P.B.); a.latorre@ucl.ac.uk (A.L.); 9 Department of Medical Sciences and Public Health, University of Cagliari, 09124 Cagliari, Italy

**Keywords:** CASPR2, peripheral nerve hyperexcitability, neuromyotonia, afterdischarges, clinical neurophysiology

## Abstract

Contactin-associated protein-like 2 (CASPR2) is a transmembrane protein of the neurexin superfamily, essential for clustering voltage-gated potassium channels, particularly Kv1, at the juxtaparanodal regions of myelinated axons. This precise localisation is essential for maintaining normal axonal excitability and preventing aberrant signal propagation. Autoantibodies targeting CASPR2 have been associated with various neurological syndromes, notably peripheral nerve hyperexcitability (PNH), which presents clinically with neuromyotonia and myokymia. PNH is characterised by distinctive electrophysiological findings, including neuromyotonic discharges, myokymic discharges, and afterdischarges, which provide diagnostic value and insight into underlying pathophysiology. This review explores the mechanisms of anti-CASPR2-associated PNH, focusing on how antibody-mediated disruption of Kv1 channel clustering leads to altered axonal excitability. Current evidence suggests that both the distal and proximal segments of the axon are sites of pathological activity, where impairments in action potential termination and re-entry prevention result in spontaneous, repetitive discharges. While afterdischarges likely originate within the axon, the precise location—whether in the alpha-motoneuron soma or axon—is uncertain. The involvement of spinal inhibitory circuits has also been proposed, though it remains speculative. Understanding the neurophysiological features of anti-CASPR2-associated PNH is essential for improving diagnostic accuracy and guiding treatment strategies. Further research is needed to clarify the mechanisms of CASPR2-related hyperexcitability.

## 1. Introduction

Recent advances in research have expanded the spectrum of neuronal antibodies and their associated neurological syndromes [1]. The association of the autoantibodies to specific disease entities, induction of disease in animal models via passive or active immunisation, and patients’ responses to immunotherapies form the basis for classifying neuronal antibodies as pathogenic [2]. While this approach is valuable from a diagnostic point of view, it does not fully explain how a specific antibody leads to the dysfunction in discrete neuronal pathways, resulting in particular neurological syndromes. Clinical neurophysiology provides a unique opportunity to explore these pathophysiological mechanisms in a patient-centric approach to the study of pathophysiological mechanisms (contrary to animal studies), particularly for antibodies that target components of the neuronal membrane.

Antibodies against contactin-associated protein-like 2 (CASPR2) have been implicated in neurological dysfunction affecting both the central and peripheral nervous systems [1,3]. Involvement of the former typically manifests as limbic encephalitis but can include complex symptoms such as cognitive impairment, seizures, dysautonomia, and insomnia [4]. Symptoms related to peripheral nerve dysfunction commonly manifest as muscle spasms, stiffness, and pain [5,6,7]. These features are broadly referred to as peripheral nerve hyperexcitability (PNH), a syndrome primarily defined by neuromyotonia and myokymia [8,9,10,11]. Over the past four decades, electrophysiological correlates of PNH have been described in various case reports and series [7,10,12], though considerable variability exists in the methodologies and techniques employed.

CASPR2, the protein encoded by the gene *CNTNAP2*, plays a crucial role in stabilising voltage-gated K^+^ channels at the juxtaparanodal region of myelinated axons. This regulation is essential for achieving the repolarisation required to terminate action potentials and restore the resting membrane potential [13]. CASPR2 antibodies may indirectly impair this process—a mechanism supported by evidence from both animal models and human studies [14,15]—and thereby contribute to the development of PNH. However, the precise mechanisms and levels at which CASPR2 dysfunction translates into this clinical syndrome remain unclear.

Neurophysiological studies offer a valuable window into the functional status of specific pathways within the nervous system [16]. Given that PNH is associated with distinct neurophysiological features, a detailed analysis of these parameters may offer important insights into the pathophysiological mechanisms underlying anti-CASPR2-associated PNH and related disorders. This narrative review aims to explore the pathophysiological basis of PNH associated with CASPR2 antibodies, with a particular focus on its neurophysiological signature. By correlating electrophysiological findings with the known actions of CASPR2 antibodies, we aim to identify dysfunctional neural pathways and advance our understanding of the disease mechanisms involved.

## 2. Functional Aspects of Voltage-Gated Potassium Channels and Related Proteins

Action potentials are initiated at the axon initial segment, a region with a high density of voltage-gated Na^+^ and K^+^ channels [17]. The coordinated opening and closure of these ion channels generate a rapid sequence of voltage changes across the neuronal membrane [18]. Specifically, the opening of Na^+^ channels allows an influx of Na^+^, leading to an all-or-nothing depolarisation that marks the onset of the action potential [19]. Subsequently, the opening of voltage-gated K^+^ (Kv) channels facilitates the efflux of K^+^, which drives axonal repolarisation and terminates the action potential [13]. In animal models, axonal whole-cell recording from layer five pyramidal cells has shown that depolarisation rapidly activates a low-threshold, slowly-inactivating, outward current mediated by Kv channels [20]. Blocking this current prolongs the action potential duration, resulting in repetitive discharges in distal axons in response to local current injection [20]. Several Kv channel subtypes have been identified, including the *Shaker* family (Kv1) [21]. An essential function of these channels lies in their inactivation properties, which allow subthreshold somatodendritic voltage changes to modulate neurotransmitter release. Under such conditions, reduced availability of Kv1 channels broadens the axonal action potential up to 1.4-fold [22]. In addition to excitatory neurons, Kv1 channels also regulate the excitability of fast-spiking neocortical GABAergic interneurons [23], and more recently, they have been implicated in controlling inhibitory circuitry within the cerebellum [24].

Kv1.1 channels are members of the Kv1 family, encoded by the *KCNA1* gene, widely expressed throughout the central and peripheral nervous system, including the neocortex, hippocampus, cerebellum, and peripheral nerves [24,25,26]. In addition to the axon’s initial segments, Kv1.1 channels are also expressed near the nodes of Ranvier of myelinated axons, where they contribute to the regulation of action potential propagation [27]. These channels are also particularly enriched in the juxtaparanodal region of axons, a specialised domain located just adjacent to the node of Ranvier but covered by myelin [14]. Both juxtaparanodal and internodal Kv1.1 channels play a crucial role play a crucial role in preventing re-entrant excitation and back-excitation [28], thereby preserving axonal stability. Using Kv1.1 knockout mice, Zhou and coworkers demonstrated that Kv1.1 channels in these myelin-covered regions become functionally more important under physiological stress conditions, such as reduced temperature [29].

**Figure 1:**
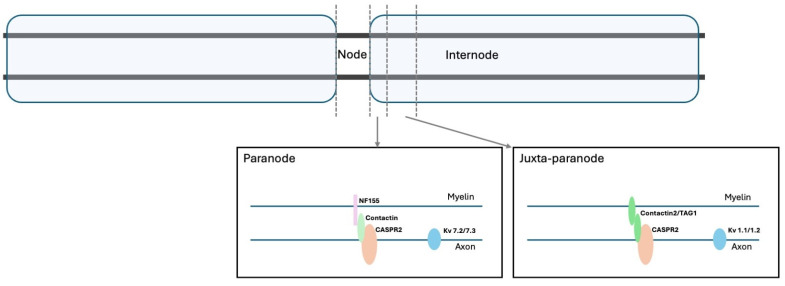
Schematic representation of VGCK and associated proteins in the paranodal and juxtaparanodal regions.

Kv1 channels are co-localised in the membrane with several cell adhesion molecules (CAMs), among which contactin-associated protein 2 (CASPR2)—a multidomain, presynaptic type 1 transmembrane protein of the neurexin superfamily [30,31]—plays a pivotal role. Its function is critical at the juxtaparanodes of myelinated axons, where it regulates K^+^ channel clustering, a process required for proper nerve impulse conduction [30]. The glycosyl-phosphatidyl-inositol-anchored CAM Transient Axonal Glycoprotein 1 (TAG-1) or Contactin-2 is necessary for the organisation of CASPR2 in the juxtaparanodal domain; without it, the normal enrichment of K^+^ channels in these regions is disrupted [32]. TAG-1, which is expressed in both neurons and myelin, may be relevant in the initial phase of Kv channel clustering [33], while CASPR2 may be involved in later stages, including the positioning and stabilisation of TAG-1/Kv1 complexes within the juxtaparanode [33]. Figure 1 shows the structural organisation of these proteins in the axonal membrane.

Further insight into Kv1 channels’ function comes from the identification of genetic defects in the *KCNA1* gene, which encodes the Kv1.1 α-subunits [34]. *KCNA1* mutations are primarily associated with a rare movement disorder known as episodic ataxia type 1 (EA1) [35,36], and have also been linked to epilepsy [35]. These clinical phenotypes are consistent with the anatomical distribution of Kv1 channels, particularly in the cerebellum and neocortex [24,25], and presumably result from faulty termination of action potentials, leading to abnormal discharges of excitatory neurons or inhibitory interneurons, resulting in paroxysms of ataxia (impaired cerebellar function) or seizures (excessive cortical activity) [37]. Interestingly, some patients with *KCNA1* mutations also exhibit signs of PNH, such as myokymia or neuromyotonia, either isolated or combined with other phenotypes [37,38,39]. Conversely, CASPR2 is coded by the *CNTNAP2* gene, located on chromosome 7. *CNTNAP2* knockout mice exhibit abnormal neuronal migration, seizures, and behavioural abnormalities—a phenotype that parallels human conditions resulting from loss-of-function mutations in this gene [40,41]. Interestingly, aside from reduced deep tendon reflexes, peripheral nerve involvement with hyperexcitability is rarely reported in this genetic condition [42]. Understanding the manifestations of these genetic disorders causing K^+^-channel dysfunction provides valuable insights into acquired conditions such as autoimmune neurological syndromes involving antibodies against components of the Kv channel complex. In the case of anti-CASPR2 autoimmunity, the convergence on shared molecular pathways helps explain the overlap between genetic and immune-mediated hyperexcitability syndromes. However, it is essential to emphasise that a genetic dysfunction may manifest in clinical phenotypes that do not entirely correlate with the dysfunction caused by an antibody-mediated attack, due to the extent and timing of the subsequent protein dysfunction.

## 3. Antibodies to Voltage-Gated Potassium Channels and Related Proteins

Located in the cell surface of neurons, voltage-gated K^+^ channels or associated proteins (VGKC-complex), such as CASPR2 and Leucine-rich, Glioma Inactivated 1 (LGI-1), are targets to circulating autoantibodies in the context of specific autoimmune neurological syndromes [1]. VGKC-complex antibodies were initially identified in patients with neuromyotonia, with the spectrum of recognised neurological syndromes expanding to the central nervous system, encompassing limbic encephalitis and cerebellar ataxia, among others [43]. PNH, which typically manifests as neuromyotonia, is considerably more common in CASPR2 compared to LGI1 autoimmunity, possibly due to the higher expression of CASPR2 in peripheral nerves compared to LGI1 [15]. When neuromyotonia occurs in combination with central nervous system symptoms such as encephalopathy, hallucinations and agrypnia excitata, the condition is referred to as Morvan syndrome [44,45], which is predominantly associated with CASPR2 antibodies. Antibody titres are generally lower in patients with isolated PNH than those with central nervous system involvement [46,47]. Interestingly, younger age has been the only factor consistently associated with isolated peripheral nerve involvement to date [48]. Furthermore, thymoma is more commonly associated with peripheral nerve involvement than with encephalitis in the context of VGKC-complex antibodies [43]. These findings suggest that peripherally generated antibodies may be sufficient to cause PNH, even at lower antibody serum concentrations [49].

Evidence supporting an autoimmune aetiology for acquired PNH stems from multiple findings: (1) the presence of oligoclonal bands in the cerebrospinal fluid in occasional cases; (2) clinical improvement following plasma exchange; (3) co-occurrence of acquired neuromyotonia with thymoma and myasthenia gravis in some cases [50,51]. Additional support for an autoimmune mechanism comes from the observation that immunoglobulin G from affected patients transferred to mice significantly enhances the resistance to d-tubocurarine at the neuromuscular junction compared to controls [52]. Moreover, intracellular microelectrode recording from diaphragm preparations showed a significantly increased neurotransmitter release at the neuromuscular junction, suggesting a presynaptic site of action, likely affecting K^+^ channels [52]. Using a patch–clamp (or whole-cell clamp) technique to record currents across ion channels, it has been shown that cells exposed to serum from patients with neuromyotonia have a suppression of K^+^ currents with normal Na^+^ conductance, supporting this pathophysiological mechanism [53,54,55].

The cellular basis for VGKC-antibody-mediated syndrome has been further clarified through immunostaining studies of mouse neural tissues using patients’ sera. Kleopas and colleagues showed that most patients’ sera bound to juxtaparanodal regions of myelinated axons, co-localising with Kv1.1 and Kv1.2 [56]. Irani and coworkers demonstrated that only a minority of patients harboured antibodies to the Kv1 subunits themselves. These were often directed against intracellular epitopes and, as a result, most likely lack pathogenic potential [57]. Instead, most antibodies target two proteins that are part of the VGKC complexes, specifically CASPR2 and LGI1 [58]. CASPR2 antibodies were more common in cases with PNH, while LGI1 antibodies were more frequently associated with limbic encephalitis [58]. CASPR2 antibodies from patient sera also showed specific reactivity with brain and peripheral nerve tissue, co-localizing with CASPR2 in transfected cells and showing no binding in tissue from CASPR2-null mice [59,60].

## 4. Neurophysiological Aspects of Peripheral Nerve Hyperexcitability in CASPR2 Autoimmunity

**Figure 2:**
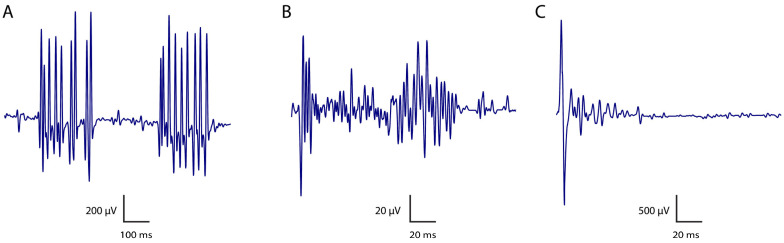
Diagram depicting classical neurophysiological features of PNH: (**A**)—myokimia; (**B**)—neuromyotonia; (**C**)—afterdischarges following a compound muscle action potential.

PNH is a term variably describing a range of neuromuscular hyperexcitability symptoms and signs that originate at the level of peripheral nerves. It is primarily characterised by spontaneous and continuous motor unit activity and slow muscle relaxation [61], and encompasses various electrophysiological phenomena [62]. The paradigmatic form of acquired PNH is that associated with CASPR2 antibodies [63], where myokymia and neuromyotonia are the predominant clinical features. A recent extensive review has clarified the terminology and potential pitfalls related to neuromuscular hyperexcitability syndromes [16], while Table 1 and Figure 2 provide a summary of the key neurophysiological features of PNH.

Given that neuromyotonia and myokymia are hallmark features of PNH in CASPR2 autoimmunity, two key questions arise: (1) to what extent do CASPR2 antibodies contribute to the pathogenesis of these symptoms? and (2) what is the precise anatomical origin of the discharges underlying neuromyotonia and myokymia? Insights from the electrophysiological properties of these abnormal discharges provide valuable clues in addressing both questions.

Mice with *KCNA1* mutations display spontaneous EMG activity in the hindlimbs compatible with myokymia [64]. This, together with the aforementioned role of juxtaparanodal Kv1 channels in maintaining axonal excitability [29], supports the hypothesis that CASPR2-induced PNH results from a failure of normal mechanisms of re-entrant excitation. In this mice model, impaired hyperpolarisation leaves axonal membranes in a persistently depolarised state and closer to the threshold for action potential generation [64]. However, this mechanism alone cannot fully explain the generation of spontaneous action potentials in the absence of external stimuli. Maddison and colleagues demonstrated a significantly prolonged strength-duration time constant in motor axons of patients with PNH compared to controls [65]. This suggests upregulation of persistent Na^+^ conductances, which may drive ectopic axonal firing in neuromyotonia [9]. This represents an indirect consequence of K^+^ channel dysfunction, while the direct effect may be insufficient hyperpolarisation, allowing re-excitation of previously activated axons and generation of consecutive bursts [66].

Supporting this, a study on human peripheral nerves obtained during graft surgery showed that slow K^+^ conductance is required to limit the repetitive response to prolonged sustained depolarisation, even though it has minimal impact on the amplitude of the action potential [67]. One hallmark of neuromyotonic discharges is a decrement in amplitude during the burst. Initially, this was attributed to the muscle fibres’ inability to sustain high-frequency input [12]. However, a study using high-density surface EMG and interspike interval (ISI) analysis in neuromyotonia showed that ISI increases linearly within each burst [68], a pattern also seen when slow potassium currents are pharmacologically blocked. In such cases, the axon remains in a supernormal state, prone to sustained discharges [67]. This suggests that the declining amplitude in neuromyotonia reflects the recovery cycle of myelinated axons, and it is dependent on slow K^+^ conductances [68].

Another prominent electrophysiological feature of anti-CASPR2-associated PNH is the presence of afterdischarges [69,70,71,72,73]. These are long, polyphasic, large-amplitude bursts observed with suprathreshold stimulation of motor axons, sufficient to obtain compound muscle action potentials (CMAP) and F waves. Similar afterdischarges may also occur following the H reflex [74,75,76]. Due to the higher gain settings (100–200 μV/division), afterdischarges may be initially noted during F wave recording. However, prolonged afterdischarges might be confused with F waves. Adjusting the gain settings may allow for better visualisation of afterdischarges during motor nerve conduction studies.

A recent study suggested a relationship between the pattern of afterdischarges and specific forms of PNH: afterdischarges following a CMAP with an absent F wave are more common in Morvan syndrome, whereas non-Morvan patients and antibody-negative patients had afterdischarges following a preserved F wave [73]. Interestingly, long-duration afterdischarges following F waves have been reported in seronegative PNH cases, suggesting a different pathophysiological mechanism [73]. While afterdischarges may be a more sensitive neurophysiological marker of PNH than other features, this observation is based on a limited number of patients [70].

Afterdischarges are believed to reflect K^+^ channel dysfunction, resulting in a failure to terminate the depolarising cascade after a motor impulse. Their latency is similar to that of F waves, and they are observed in both distal and proximal muscles [77]. This latency, along with their repetitive nature, supports a model involving antidromic activation of anterior horn cells that then triggers repetitive motor discharges indistinguishable from F waves [70,77]. The duration of afterdischarges also appears to increase with higher stimulation frequencies [78], consistent with impaired Kv1.1 function. Taken together, these findings suggest that although an initial external stimulus may be required to initiate neuromyotonic discharges, sustained activity does not depend on continued external input.

The recovery cycle of human motor axons comprises an absolute and relative refractory period, followed by a period of supernormal phase [79,80]. Repetitive neuromyotonic discharges mirror this pattern, as a second train of impulses can only be triggered after a first one with a delay of 3.5 to 4 ms, just above the refractoriness of the synapse-muscle fibres [81]. Interestingly, the refractory period can be longer when considering specifically triplet and quadruplet motor unit discharges, with an interval of 30 ms between the first discharge of a triplet and a second triplet having been reported [7]. One study also investigated the afterdischarge recovery by paired stimulation in a patient with diabetic neuropathy and myokymia [77]. The refractory period of these late repetitive responses ranged from 100 and 300 ms [77]—durations too long to be explained solely by axonal properties. The authors proposed that changes in anterior horn cell excitability might contribute, although this mechanism may not be relevant in CASPR2-mediated PNH.

There is compelling evidence that the ectopic discharges in neuromyotonia originate in the peripheral nerve. Supporting observations include the persistence of spontaneous motor unit activity during sleep and general anaesthesia, the resolution of abnormal movements with pharmacological blockade of peripheral nerves, and the abolition of abnormal discharges by intramuscular injection of botulinum toxin [6,10,11,82]. Moreover, one study by Vucic and colleagues showed normal cortical inhibition assessed by paired-pulse transcranial magnetic stimulation in patients with acquired neuromyotonia [83], arguing against a central contribution to PNH. Taken together, these findings favour a peripheral origin for the ectopic discharges in neuromyotonia, likely driven by secondary changes in Na^+^ conductances resulting from Kv channel dysfunction [65]. However, no studies have yet investigated spinal or brainstem reflexes in anti-CASPR2-associated neuromyotonia, which could help determine whether inhibitory central circuits are also involved.

## 5. Discussion

CASPR2-associated disorders can present with a variety of clinical manifestations, yet the reasons behind this clinical heterogeneity remain poorly understood [1,2]. CNS involvement in some patients with CASPR2 autoantibodies reflects the broad expression of this protein in the CNS [31]. Still, it remains unclear why certain individuals with serum anti-CASPR2 antibodies and intact peripheral nerve targets develop exclusively CNS manifestations, without signs of peripheral nerve dysfunction [15]. Conversely, PNH often appears as an isolated syndrome, offering a distinct opportunity to explore the underlying pathophysiological mechanisms of CASPR2-related neurological dysfunction.

Within this context, the diverse electrophysiological findings in PNH may offer important insights into the pathophysiological effects of anti-CASPR2 antibodies. Characteristic findings such as neuromyotonic and myokymic discharges, as well as afterdischarges, not only aid in diagnosis but may also reflect specific disruptions in axonal excitability caused by CASPR2 autoantibodies. However, despite the value of these techniques, current data specifically addressing anti-CASPR2-associated PNH remain limited [5]. This is mainly due to the heterogeneity in study cohorts, which often combine neuromyotonia of varying aetiologies and lack standardised neurophysiological protocols [9,10]. Moreover, analyses of afterdischarges and recovery cycles have frequently included patients with ambiguous or unconfirmed antibody status, which complicates interpretation [7,58,65,77].

The CASPR2-mediated hyperexcitability of peripheral nerves appears to result from the disruption of axonal K^+^ channel function, driven by autoantibodies targeting CASPR2 and interfering with its role in clustering Kv1 channels at juxtaparanodes. This hypothesis is supported by in vitro studies, animal models, and clinical reports of patients with genetic mutations in the involved pathways [32,59]. However, the precise anatomical origin of the abnormal discharges in anti-CASPR2-associated PNH has not been definitively established.

Most of the available evidence points toward a peripheral origin for ectopic activity in anti-CASPR2-associated neuromyotonia. Yet, the precise site of origin—distal versus proximal segments of the nerve—remains a matter of debate. Afterdischarges, which are relatively specific for PNH, are more commonly observed in the tibial nerve compared to more proximal segments [84,85,86]. This may reflect the anatomical distribution of hyperexcitability and the increased vulnerability of distal nerves. For instance, VGKC mediates fast K^+^ repolarising in distal motor nerve terminals, where their dysfunction may allow re-entrant excitation [5,87]. At these sites, the axons are poorly insulated by the blood-nerve barrier or myelin sheath and are potentially vulnerable to circulating autoantibodies [88]. This distal vulnerability may explain why the abnormal discharges are more readily recorded distally in some studies [84,87]. Moreover, spontaneous discharges originating at the distal axon or motor endplate may become self-sustaining due to impaired Kv1.1-mediated re-entry control, further contributing to persistent hyperexcitability in anti-CASPR2-associated PNH [87,89]. Another possible explanation is the degree of PNH in itself. Induction of membrane hyperexcitability following electrical stimulation facilitates the sustained appearance of afterdischarges, contrasting with states of relatively low PNH in which abnormal spontaneous discharges might not be evident on resting needle EMG [70].

However, the possibility that proximal nerve segments are involved cannot be excluded. Data on synchronicity of spontaneous discharges obtained by analysing time-locked fasciculations in double recordings suggest that abnormal discharges may originate from both proximal and distal branches [90]. Furthermore, studies using multilevel nerve blocks to suppress continuous muscle fibre activity point to proximal origins, and in some cases, dual generators above and below the block site were proposed [91]. In line with this, the latency of afterdischarges generated after suprathreshold motor axon stimulation is compatible with antidromic activation of spinal motor neurons [69]. Considering the high expression of VGKC and associated proteins in the initial segment of motor axons [17,21], this site could plausibly contribute to abnormal impulse initiation in the presence of anti-CASPR2.

A central generator for neuromyotonia appears unlikely, as PNH does not improve with reductions of central activity [6,10,11,82,83]. Nevertheless, the potential role of spinal mechanisms cannot be entirely excluded. CASPR2 is expressed in multiple neuron types across the nervous system [24,92], and the involvement of spinal interneurons at a local level or indirect involvement of other descending pathways has not been previously studied in PNH. Furthermore, the distinctive phenomenon of afterdischarges could point to the contribution of the alpha-motoneuron soma in PNH.

**Figure 3:**
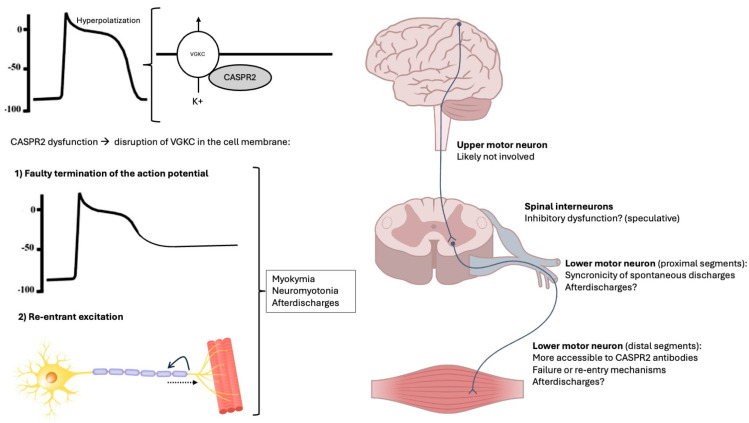
Illustration of the proposed pathophysiological implications of CASPR2 in peripheral nerve hyperexcitability and location of the generator in the nervous system.

Notably, spinal myoclonus has been reported in association with anti-CASPR2 autoantibodies, even in the absence of neuromyotonia [93,94]. The mechanisms of spinal myoclonus remain speculative and may involve impaired inhibition in spinal circuits, hyperactivity of alpha-motoneurons, aberrant axonal re-excitation, and/or dysfunctional inhibition from descending pathways [95,96]. Thus, it is possible that anti-CASPR2 also affects local spinal inhibitory circuits. However, despite some evidence that the H-reflex might induce afterdischarges in CASPR2 antibody-associated neuromyotonia [76], spinal inhibitory reflexes have never been studied in the context of PNH associated with anti-CASPR2. However, given that co-contraction of antagonistic muscles—a hallmark of impaired reciprocal inhibition—is not a feature of neuromyotonia [10], spinal inhibitory dysfunction is likely not a major contributor. Furthermore, although it is known that central inhibitory interneurons express CASPR2 [97], its expression in Renshaw cells and other spinal inhibitory interneurons has not been studied in depth. Studying reciprocal inhibition of the H-reflex in neuromyotonia would help clarify the contribution of inhibitory dysfunction to the pathophysiology of anti-CASPR2. Figure 3 illustrates the proposed mechanisms and sites of abnormal activity generation.

The neurophysiological features of PNH appear to be the same irrespective of the antibody status [77,78,84]. Some patients may be negative for CASPR2 or other antibodies and display a clinical phenotype compatible with PNH. Neurophysiological testing may be useful in confirming the diagnosis in these cases and potentially pave the way for the discovery of novel antibodies, as well as understanding how they interfere with the dynamics of membrane excitability. Conversely, alternative disease mechanisms may be involved in these forms of PNH.

From a neurophysiological perspective, it is instructive to compare anti-CASPR2 neuromyotonia with stiff person spectrum disorders (SPSDs). While both conditions feature continuous motor unit activity, their pathophysiological underpinnings differ: in neuromyotonia, hyperexcitability originates in the peripheral nerve, whereas in SPSD, reduced central inhibition results in disinhibited spinal motor output [98]. This distinction explains the presence of reciprocal inhibition abnormalities and pathological startle responses in SPSD but not in neuromyotonia. Although some CNS GAD65-positive inhibitory neurons (commonly implicated in SPSD) express high levels of CASPR2 [1,97], anti-CASPR2 antibodies likely do not impair inhibitory function to the extent seen in SPSD. Future comparative studies of neurophysiological profiles in PNH and SPSD may further illuminate the differential contributions of central and peripheral mechanisms.

## 6. Conclusions

Anti-CASPR2 antibodies are associated with PNH, manifesting as neuromyotonia and myokymia. CASPR2 plays a critical role in the optimal disposition of VGKC along axonal membranes, and its disruption by autoantibodies leads to abnormal nerve excitability phenomena. Neurophysiological hallmarks such as neuromyotonic and myokymic discharges, along with afterdischarges, provide relevant information on pathophysiological aspects of PNH. Current evidence suggests that the axon, both distally and proximally, is the primary site of pathological activity, with dysfunctions in action potential termination and re-entry prevention being central mechanisms. While the role of spinal inhibitory circuits remains speculative, ongoing research is essential to fully elucidate the pathophysiological underpinnings of CASPR2-associated PNH and refine diagnostic and therapeutic approaches.

## Figures and Tables

**Table 1:** Summary of the key neurophysiological features of PNH.

PNH Feature	Definition	Electrophysiological Features
**Myokymia**	Random, undulating, rippling muscle movements (from the Greek *kymia*, which means wave). Typically recur rhythmically or semirhythmically and may range from focal (most commonly in facial muscles) to generalised.	Spontaneously generated bursts of single motor unit action potentials firing at 5–150 Hz rates. May occur as repetitive doublets, triplets, or multiplets, with interburst frequencies 1–5 Hz. Distinguished from fasciculations by its rhythmicity and involvement of the same motor units in each discharge. Characteristic “marching soldiers” sound in routine EMG, where changing to a longer sweep speed during recording makes it easier to recognise the bursting pattern of myokymic discharges. Freezing the screen often makes it easier to recognise the presence of the same motor unit potential firing repetitively in bursts.
**Neuromyotonia**	Also referred to as Isaac syndrome, pseudomyotonia, neurotonia and normo-calcemic tetany. Generalised muscle stiffness, delayed muscle relaxation and excessive sweating (hyperhidrosis). Muscle taping does not trigger a myotonic discharge as in myotonia.	Spontaneous, high-frequency and sustained motor unit discharge firing at 150–300 Hz, manifesting as prolonged bursts lasting up to a few seconds, with an abrupt onset and termination. Originates from motor neurons or their axons (in contrast with muscle fibres, as seen in myotonia). There is no evidence that myokymic and neuromyotonic discharges are distinct phenomena arising from different mechanisms. The higher frequency of neuromyotonic discharges may simply reflect the longer duration of their bursts. Characteristic “pinging” sound in routine EMG, where changing the sweep speed allows the identification of each potential as the same motor unit action potential.

## Data Availability

No new data were created or analysed in this study. Data sharing is not applicable to this article.

## Abbreviations

The following abbreviations are used in this manuscript:

CAM	Cell adhesion molecules
CASPR2	Contactin-associated protein-like 2
LGI1	Leucine-rich, Glioma Inactivated 1
PNH	Peripheral nerve hyperexcitability
SPSD	Stiff person spectrum disorders
TAG-1	Transient Axonal Glycoprotein 1 (TAG-1)
VGKC	Voltage-gated potassium channel
